# Anti-Melanogenic and Anti-Inflammatory Effects of 2′-Hydroxy-4′,6′-dimethoxychalcone in B16F10 and RAW264.7 Cells

**DOI:** 10.3390/cimb46060359

**Published:** 2024-06-14

**Authors:** Sungmin Bae, Jung-No Lee, Chang-Gu Hyun

**Affiliations:** 1 Department of Beauty and Cosmetology, Jeju Inside Agency and Cosmetic Science Center, Jeju National University, Jeju 63243, Republic of Korea; 9901bae99@naver.com; 2 Bio Convergence R&D Center, CoSeedBioPharm Corporation, Heungdeok-gu, Cheongju 28161, Republic of Korea; rnd@coseed.co.kr

**Keywords:** anti-inflammation, anti-melanogenesis, B16F10, 2′-hydroxy-4′,6′-dimethoxychalcone, RAW 264.7

## Abstract

Chalcone is a type of flavonoid compound that is widely biosynthesized in plants. Studies have shown that consuming flavonoids from fruits and vegetables or applying individual ingredients reduces the risk of skin disease. However, the effects of chalcone on melanogenesis and inflammation have not been fully investigated. The aim of this study was to evaluate the anti-melanogenic and anti-inflammatory effects of 2′-hydroxy-3,4′-dimethoxychalcone (3,4′-DMC), 2′-hydroxy-4,4′-dimethoxychalcone (4,4′-DMC), 2′-hydroxy-3′,4′-dimethoxychalcone (3′,4′-DMC), and 2′-hydroxy-4′,6′-dimethoxychalcone (4′,6′-DMC). Among the derivatives of 2′-hydroxy-4′-methoxychalcone, 4′,6′-DMC demonstrated the most potent melanogenesis-inhibitory and anti-inflammatory effects. As evidenced by various biological assays, 4′,6′-DMC showed no cytotoxicity and notably decreased the expression of tyrosinase, tyrosinase-related protein (TRP)-1, and TRP-2 enzymes. Furthermore, it reduced cellular melanin content and intracellular tyrosinase activity in B16F10 melanoma cells by downregulating microphthalmia-associated transcription factor (MITF), cAMP-dependent protein kinase (PKA), cAMP response element-binding protein (CREB), p38, c-Jun N-terminal kinase (JNK), β-catenin, glycogen synthase kinase-3β (GSK3β), and protein kinase B (AKT) proteins, while upregulating extracellular signal-regulated kinase (ERK) and p-β-catenin. Additionally, treatment with 4′,6′-DMC significantly mitigated the lipopolysaccharide (LPS)-induced expression of NO, PGE_2_, inflammatory cytokines, COX-2, and iNOS proteins. Overall, 4′,6′-DMC treatment notably alleviated LPS-induced damage by reducing nuclear factor kappa B (NF-κB), p38, JNK protein levels, and NF-kB/p65 nuclear translocation. Finally, the topical applicability of 4′,6′-DMC was evaluated in a preliminary human skin irritation test and no adverse effects were found. These findings suggest that 4′,6′-DMC may offer new possibilities for use as functional ingredients in cosmeceuticals and ointments.

## 1. Introduction

Human skin color results primarily from pigments like melanin (black, brown, or red), carotene (yellow), and hemoglobin (red) present in the skin. These pigments’ expression is regulated by environmental factors such as UV radiation, fatigue, stress, hormones, and genetic factors, with melanin being the most influential [1,2,3,4,5]. Melanin, found in the skin, hair, and eyes, determines skin color [6,7]. Synthesized in melanocytes, pigmented cells in the basal epidermal layer, melanin is stored in melanosomes, organelles within melanocytes [8,9]. Melanosomes produce and transfer melanin to surrounding keratinocytes, which determines an individual’s skin color [10,11,12,13]. Melanin serves a vital role in shielding the body from harmful UV radiation by absorbing UV rays and regulating the skin’s immune response. However, excessive melanin production and accumulation in the skin can lead to hyperpigmentation disorders such as melasma, freckles, and age spots [14,15,16,17,18].

When keratinocytes in the skin are exposed to external stimuli like UV light, they release intracellular α-melanocortin-stimulating hormone (α-MSH) [19,20]. This released α-MSH then signals melanocytes to activate various signaling pathways, including mitogen-activated protein kinases (MAPK), cyclic adenosine monophosphate (cAMP)/protein kinase A (PKA), phosphoinositide 3-kinase (PI3K)/protein kinase B (AKT), and Wnt/β-catenin, promoting melanin production [21,22]. Microphthalmia-associated transcription factor (MITF), a key transcription factor activating the expression of melanogenic genes, stimulates the production of tyrosinase (TYR), tyrosinase-related protein (TRP)-1, and TRP-2 proteins, thereby regulating melanogenesis [23,24,25]. Consequently, inhibiting the activity of pivotal enzymes and regulators like MITF, TYR, TRP-1, and TRP-2, along with various melanin-producing signal transduction pathways, is deemed a critical approach in developing melanogenesis inhibitors.

Inflammation is a ubiquitous physiological response in the human body, serving as a vital defense mechanism against external threats [26]. However, when inflammation becomes excessive, it can precipitate severe inflammatory ailments affecting human skin, including eczema, seborrheic dermatitis, acne, folliculitis, allergic urticaria, and psoriasis [27,28]. Lipopolysaccharide (LPS), a key constituent of the outer membrane of Gram-negative bacteria, exerts significant regulatory control over inflammation [29,30]. Consequently, LPS-stimulated macrophages serve as a valuable model for investigating inflammation and exploring the mechanisms underlying potential anti-inflammatory interventions [31].

Macrophages, via Toll-like receptors (TLRs) on their surface, identify pathogens and instigate the production of pro-inflammatory cytokines like interleukin (IL)-6, IL-1β, and TNF-α, along with reactive oxygen species (ROS) generated by inducible nitric oxide synthase (iNOS) and cyclooxygenase-2 (COX-2). These agents play pivotal roles in inflammatory conditions characterized by excessive inflammatory mediator release, such as nitric oxide (NO) and prostaglandin E_2_ (PGE_2_) [32,33,34]. The synthesis of inflammatory mediators in macrophages is mediated by TLR4, among other TLRs, triggered by LPS [35]. Consequently, suppressing the generation of these inflammatory mediators emerges as a critical objective in managing inflammatory disorders. Upon LPS stimulation, nuclear factor kappa B (NF-kB) translocates from the cytoplasm to the nucleus, regulating the expression of iNOS and COX-2 genes to modulate inflammatory mediator production [36,37]. Furthermore, the MAPK pathway can prompt the expression of pro-inflammatory genes, encompassing interleukins, tumor necrosis factor-alpha (TNF-α), iNOS, and COX-2 [38,39]. Thus, the NF-κB and MAPK pathways represent pivotal targets for therapeutic interventions in diverse inflammatory conditions.

Chalcones, characterized by their α-β-unsaturated ketone structure linking two aromatic rings, are commonly found in both edible and medicinal plant compounds, imparting a yellow hue [40]. Their straightforward chemical makeup and ease of synthesis have led to the preparation of numerous chalcone derivatives, which exhibit diverse activities across various biological systems. These derivatives have demonstrated efficacy in fields such as antiparasitic, antioxidant, anti-inflammatory, neuroprotective, antiulcer, and antifungal activities [41,42,43,44]. With ongoing success stories regarding their synthesis and biological evaluation, there is a burgeoning interest in chalcone analogs and their potential medicinal applications [45]. Consequently, the promising biological profiles of chalcone derivatives have garnered significant attention from both academia and industry. Chalcone, a secondary metabolite belonging to the flavonoid (C6-C3-C6 system) family, is widely distributed in both edible and medicinal plants. Chalcone and its natural derivatives serve as crucial intermediates in the flavonoid biosynthetic pathway [46]. The role of methoxy groups in flavonoids and chalcone structures is a topic of debate; while their contribution to anti-inflammatory properties is generally acknowledged, their impact on melanogenesis is controversial. Notably, nobiletin and tangeretin, representative methoxy flavonoids, stimulate melanogenesis in B16F10 melanoma cells [47,48]. Conversely, our previous study demonstrated the inhibitory effect of 2′-hydroxy-3,6′-dimethoxychalcone on melanogenesis in the same cells and under similar conditions [49]. Therefore, further investigations into methoxy chalcones are imperative to definitively elucidate the role of the methoxy group in chalcone structures.

**Figure 1:**
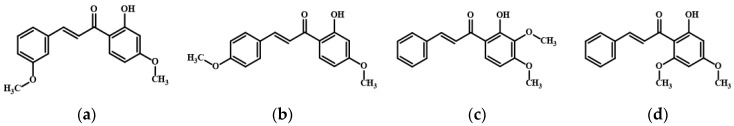
The chemical structures of (**a**) 3,4′-DMC, (**b**) 4,4,’-DMC, (**c**) 3′,4′-DMC, and (**d**) 4′,6′-DMC.

During our ongoing screening program aimed at repurposing classical drugs and natural compounds, we have previously reported the anti-inflammatory and melanogenic activities of several antibiotics, flavonoids, and coumarins, which hold potential applications in cosmeceuticals and nutraceuticals [50,51,52,53]. Expanding upon this research, we conducted a screening of various 2′-hydroxy-4′-methoxychalcone derivatives, characterized by a structurally simple design, to delineate the structural attributes contributing to the bioactivities within this chemical class. In this study, we assessed the impact of 2′-hydroxy-3,4′-dimethoxychalcone (3,4′-DMC), 2′-hydroxy-4,4′-dimethoxychalcone (4,4′-DMC), 2′-hydroxy-3′,4′-dimethoxychalcone (3′,4′-DMC), and 2′-hydroxy-4′,6′-dimethoxychalcone (4′,6′-DMC) on inflammation and melanogenesis in mouse B16F10 melanoma and macrophage RAW 264.7 cells (Figure 1). Additionally, we elucidated the mechanism of action underlying the most potent derivative, 4′,6′-DMC.

## 2. Materials and Methods

### 2.1. Chemicals and Antibodies

3,4′-DMC, 4,4′-DMC, and 3′,4′-DMC utilized in this study were purchased from Sigma-Aldrich (St. Louis, MO, USA), while 4′,6′-DMC was obtained from ChemFaces (Wuhan, China). Dulbecco’s modified Eagle medium (DMEM) and penicillin/streptomycin (P/S) were procured from Thermo Fisher Scientific (Waltham, MA, USA), while fetal bovine serum (FBS) was sourced from Merck Millipore (Burlington, MA, USA). 10X Trypsin-EDTA (0.5%) was also acquired from Thermo Fisher Scientific (Waltham, MA, USA). LPS from *Escherichia coli*, Griess reagent (1% sulfanilamide, 0.1% N-(1-naphthyl) ethylenediamine, 2.5% phosphoric acid), α-MSH, NaOH, L-3,4-dihydroxyphenylalanine (L-DOPA), and protease/phosphatase inhibitor cocktail were purchased from Sigma-Aldrich (St. Louis, MO, USA). 3-(4,5-dimethylthiazol-2-yl)-2,5-diphenyltetrazolium bromide (MTT), dimethyl sulfoxide (DMSO), phosphate-buffered saline (PBS), and radioimmunoprecipitation assay (RIPA) buffer were obtained from Biosesang (Seongnam, Gyeonggi-do, Republic of Korea). Additionally, L-N6-(1-iminoethyl)lysine dihydrochloride (L-NIL) as a control was procured from Cayman Chemical Company (Ann Arbor, MI, USA), and arbutin was purchased from Sigma-Aldrich (St. Louis, MO, USA). Enzyme-linked immunosorbent assay (ELISA) kits for measuring pro-inflammatory cytokines, namely IL-6, IL-1β, and TNF-α, were acquired from BD Biosciences (Franklin Lakes, NJ, USA). The bicinchoninic acid (BCA) protein kit for protein quantification and NE-PER™ nuclear and cytoplasmic extraction reagents for nuclear isolation were sourced from Thermo Fisher Scientific (Waltham, MA, USA). 2 × Laemmli sample buffer and Tween 20 used for Western blot experiments were obtained from Bio-Rad (Hercules, CA, USA). Skim milk was acquired from BD Difco (Sparks, MD, USA), and bovine serum albumin (BSA) was obtained from Bovostar (Bovogen, Melbourne, Australia). 2-Mercaptoethanol was purchased from Biobasic (Markham, ON, Canada). Sodium dodecyl sulfate (SDS), tris-buffered saline (TBS), and enhanced chemiluminescence (ECL) kit were acquired from Biosesang (Seongnam, Gyeonggi-do, Republic of Korea). Among the primary antibodies used for Western blot experiments, TYR, TRP-1, TRP-2, MITF, p-CREB, CREB, and β-actin were purchased from Santa Cruz Biotechnology (Dallas, TX, USA), while p-GSK-3β, GSK-3β, p-β-catenin, β-catenin, p-AKT, AKT, p-ERK, ERK, p-JNK, JNK, p-p38, p38, p-PKA, PKA, p-IκB-α, IκB-α, β-actin, p65, and lamin B, as well as secondary antibodies anti-mouse and anti-rabbit, were acquired from Cell Signaling Technology (Danvers, MA, USA). Furthermore, anti-iNOS antibody was purchased from Merck Millipore (Burlington, MA, USA), and anti-COX-2 antibody was obtained from BD Biosciences (Franklin Lakes, NJ, USA).

### 2.2. Cell Viabilities

The cell viability of samples towards the RAW 264.7 macrophages was performed applying the MTT assay, one of the most widely used methods for analyzing cell proliferation and viability. The MTT method relies on the breakdown of the yellow, water-soluble MTT tetrazolium and its reduction by mitochondrial enzymes into the purple, non-water-soluble MTT formazan. During MTT exposure, the amount of formazan produced is directly proportional to the number of living cells present. Briefly, RAW 264.7 cells were seeded at 1.5 × 10^5^ cells/well in 24-well plates and preincubated for 24 h in a 37 °C, 5% CO_2_ incubator. They were then treated with the sample and LPS (1 μg/mL) and incubated under the same culture conditions for 24 h. Subsequently, they were treated with 0.2 mg/mL MTT reagent and incubated for 3 h. The MTT reagent-treated medium was removed, and the purple formazan crystals formed by reduction were dissolved using 800 μL of DMSO per well. The dissolved solution was then transferred to a 96-well plate in 100 μL aliquots, and the absorbance was measured at 570 nm using a microplate spectrophotometer reader (Epoch BioTek, Winooski, VT, USA). To determine cell viability for the whitening efficacy assay, B16F10 cells were seeded in 24-well plates at 8.0 × 10^3^ cells/well and preincubated in a 37 °C, 5% CO_2_ incubator for 24 h. Then, the samples were treated and incubated under the same culture conditions for 72 h. They were then treated with 0.2 mg/mL MTT reagent and incubated for 3 h. The MTT reagent-treated medium was removed, and the purple formazan crystals formed during the reduction were dissolved using 500 μL of DMSO per well. The dissolved solution was transferred to a 96-well plate in 100 μL aliquots, and the absorbance was measured at 570 nm using a microplate spectrophotometer reader (Epoch BioTek, Winooski, VT, USA).

### 2.3. Measurement of Nitric Oxide (NO) Production

The protective effect of the sample on LPS-induced RAW 264.7 cells was assessed following a previously reported method [51,53]. In brief, RAW 264.7 cells were seeded at 1.5 × 10^5^ cells/well in 24-well plates and incubated for 24 h at 37 °C with 5% CO_2_. After treating the samples with various concentrations ensuring above 90% cell viability, all wells except for the control were treated with 1 μg/mL LPS. During this process, 40 μM L-NIL was used as a positive control, and the samples were treated and allowed to react for 24 h. Subsequently, in a 96-well plate, 100 μL each of the treated cell cultures and Griess reagent were mixed at a 1:1 ratio and incubated for 10 min, after which the absorbance was measured at 540 nm using a microplate spectrophotometer reader (Epoch BioTek, Winooski, VT, USA).

### 2.4. Measurement of Pro-Inflammatory Cytokine Production

When exposed to inflammatory stimuli, macrophages secrete cytokines such as IL-1β, IL-6, and TNF-α. Hence, to determine whether the sample inhibits the production of pro-inflammatory cytokines, which are mediators of the inflammatory response, in LPS-induced RAW 264.7 macrophages, we employed an enzyme-linked immunosorbent assay (ELISA). RAW 264.7 cells were seeded at 1.5 × 10^5^ cells/well in 24-well plates and pre-incubated at 37 °C with 5% CO_2_ for 24 h. The samples were treated with various concentrations ensuring cell viability above 90%, and all wells except for the control were treated with 1 μg/mL LPS and incubated for 24 h. After incubation, the cell culture in each well was centrifuged at 15,000 rpm for 20 min to obtain a supernatant. This supernatant was stored at −20 °C and subsequently used to measure the content of IL-1β using aliquots and IL-6 and TNF-α using dilutions of the cell culture, following the manufacturer’s instructions for the ELISA kit.

### 2.5. Measurement of Melanin Contents

B16F10 cells were seeded at 5 × 10^4^ cells/dish in 60 mm cell culture dishes and incubated at 37 °C with 5% CO_2_ for 24 h. Subsequently, the cells were treated with 100 nM α-MSH at various concentrations ensuring above 90% cell viability and allowed to incubate for 72 h. The control group received no treatment, the negative control was treated with 100 nM α-MSH only, and the positive control was treated with 200 μM arbutin. Following the 72 h incubation period, the medium was removed, and the cells were washed twice with 1× PBS buffer. Subsequently, the cells were lysed in a buffer comprising RIPA buffer and 1% protease inhibitor cocktail for 20 min at 4 °C using 150 μL in each dish. The lysate was then transferred to a 1.5 mL e-tube and centrifuged (Smart R17 from Hanil Scientific Inc., Gimpo, Republic of Korea) at 4 °C and 15,000 rpm for 30 min, and the supernatant was discarded to obtain a pellet. The pellet was dissolved by adding 250 μL of 1N NaOH containing 10% DMSO for 15 min at 80 °C. This solution was then transferred to 96-well plates, with 50 μL in each well, and the absorbance was measured at 405 nm using a microplate spectrophotometer reader (Epoch BioTek, Winooski, VT, USA).

### 2.6. Measurement of Intracellular Tyrosinase Activity

To determine the inhibitory effect of the sample on tyrosinase activity, which leads to skin darkening due to the oxidation of tyrosine to dopaquinone, intracellular tyrosinase activity was measured. B16F10 cells were seeded at 5 × 10^4^ cells/dish in 60 mm cell culture dishes and incubated for 24 h at 37 °C with 5% CO_2_. Subsequently, they were treated with 100 nM α-MSH at various concentrations ensuring cell viability above 90% and allowed to incubate for 72 h. The control group received no treatment, the negative control was treated with 100 nM α-MSH only, and the positive control was treated with 200 μM arbutin. Following the 72 h incubation period, the medium was removed, and the cells were carefully washed twice with 1× PBS buffer before being lysed in a buffer comprising RIPA buffer and 1% protease inhibitor cocktail, using 150 μL in each dish, for 20 min at 4 °C. The lysates were then transferred to 1.5 mL e-tubes using a scraper and centrifuged (Smart R17 from Hanil Scientific Inc., Gimpo, Republic of Korea) at 4 °C and 15,000 rpm for 30 min to obtain the supernatant. Following the protocol of the BCA protein assay kit, 10 μL of the supernatant and 200 μL of the BCA reagent, mixed with BCA reagent A and B at a 50:1 ratio, were added to a 96-well plate and incubated at 37 °C for 30 min. The absorbance was then measured at 562 nm using a microplate spectrophotometer reader (Epoch BioTek, Winooski, VT, USA) after constructing a standardization curve with BSA to measure the protein content of the separated supernatants. The protein in each supernatant was diluted in equal amounts and mixed in a 96-well plate with 20 μL of the protein sample and 80 μL of L-DOPA (2 mg/mL) in each well. Following incubation at 37 °C for 2 h, the absorbance was measured at 490 nm using a microplate spectrophotometer reader (Epoch BioTek, Winooski, VT, USA).

### 2.7. Western Blot

Western blot experiments were conducted to assess whether the samples inhibited the expression of proteins involved in various signaling pathways associated with inflammation and melanogenesis. RAW 264.7 cells were seeded at 6.0 × 10^5^ cells/dish in 60 mm cell culture dishes and incubated at 37 °C with 5% CO_2_ for 24 h. Subsequently, cells were treated with various concentrations of the sample and 1 μg/mL LPS, ensuring cell viability above 90%, and incubated for durations specific to the expression time of the target protein. B16F10 cells were seeded at 7.0 × 10^4^ cells/dish in 60 mm cell culture dishes and incubated at 37 °C with 5% CO_2_ for 24 h. These cells were then treated with various concentrations of samples, ensuring cell viability above 90%, followed by treatment with 100 nM α-MSH, with sample treatment durations varying according to the expression time of the target protein. After incubation, the medium was aspirated, and cells were washed twice with 1× PBS buffer. Subsequently, cells were lysed using lysis buffer containing RIPA buffer and 1% protease inhibitor cocktail, using 150 μL in each dish, and incubated at 4 °C for 20 min. The lysate was then transferred to a 1.5 mL e-tube, centrifuged at 4 °C and 15,000 rpm for 30 min to obtain the supernatant. Following the BCA protein assay kit protocol, 10 μL of the supernatant and 200 μL of the BCA reagent, mixed with BCA reagent A and B at a 50:1 ratio, were added to a 96-well plate. This mixture was incubated at 37°C for 30 min, and the absorbance was measured at 562 nm using a microplate spectrophotometer reader (Epoch BioTek, Winooski, VT, USA) after constructing a standard curve with BSA to determine the protein content. Equal amounts of proteins from each supernatant were diluted and mixed with 2× Laemmli sample buffer in a 1:1 ratio and heated at 100 °C for 5 min to prepare samples for Western blot experiments. After cooling, 16 μL of the samples were loaded onto an SDS-PAGE and electrophoresis was performed to separate proteins by size. The separated proteins were then transferred to a PVDF membrane using the Trans-Blot Turbo System and blocked with 1× TBS-T containing 5% skim milk for 1.5 h. The membrane was washed six times with 1× TBS-T at 10 min intervals and incubated overnight at 4 °C with primary antibodies diluted to 1:2000 in 20 mL of 1× TBS-T. After washing, the membrane was incubated with secondary antibody diluted to 1:2000 in 1× TBS-T for 2 h at room temperature. Following additional washing, the membrane was reacted with an ECL kit to visualize specific protein bands, and each target protein band was detected using ChemiDoc (Bilvers-Lourmette, France).

### 2.8. Human Skin Patch Test

We selected 34 women (aged 41.56 ± 7.37 years) who met the inclusion criteria, from healthy men and women aged 20 to 60 years without skin disease. After 24 h of application, the test material was applied to the back area of the subjects and then removed. Subjects were examined through observation 20 min after application and 24 h after removal. The evaluation criteria were assessed in accordance with the PCPC guidelines, and the skin reaction results for each test substance were calculated using the following formula. This study was conducted in compliance with the ethical principles of medical research outlined in the Declaration of Helsinki, with approval from the IRB of Dermapro Inc. and consent obtained from each volunteer (IRB number: 1-220777-A-N-01-DICN22193).

$$\mathrm{Response}=\frac{\sum \left(Grade\times No.\;of\;Responders\right)}{4(Maximum\;Grade)\times n(Total\;Subjects)}\times 100\times 1/2$$

### 2.9. Statistical Analyses

Statistical analysis was conducted using IBM SPSS (v. 20, SPSS Inc., Armonk NY, USA) utilizing Student’s *t*-test or one-way analysis of variance. All experimental results were presented as the mean ± standard deviation (SD) derived from either at least three independent experiments or a single triplicate experiment. Statistically significant *p*-values were denoted as follows: 0.1 (*), 0.01 (**), or 0.001 (***).

## 3. Results

### 3.1. 2′-Hydroxy-4′-Methoxychalcone Derivatives Inhibited Melanin Content and Tyrosinase Activity in B16F10 Cells

**Figure 2:**
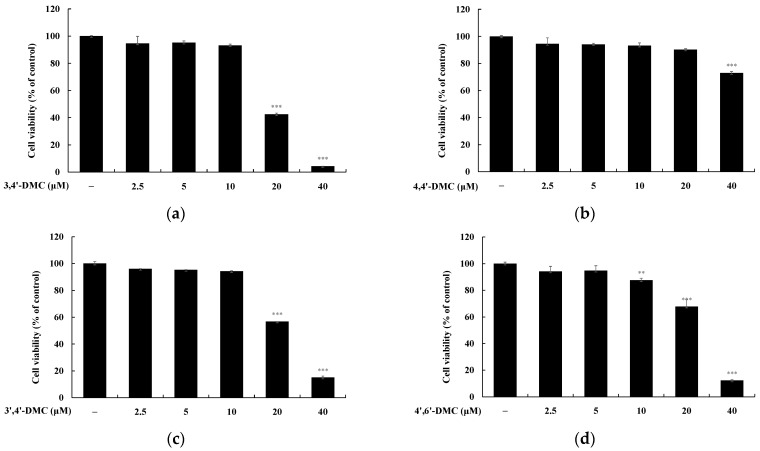
Cell viability of 2′-hydroxy-4′-methoxychalcone derivatives in B16F10 cells. The cells were treated with 2.5, 5, 10, 20, and 40 μM 2′-hydroxy-4′-methoxychalcone derivatives for 72 h. Cell viability of B16F10 cells subjected to (**a**) 3,4′-DMC, (**b**) 4,4,’-DMC, (**c**) 3′,4′-DMC, and (**d**) 4′,6′-DMC was measured using an MTT assay. Results are expressed as the mean ± SD of three independent experiments. ** *p* < 0.001, *** *p* < 0.001 compared with untreated control.

To determine the non-cytotoxic concentrations of 2′-hydroxy-4′-methoxychalcone derivatives in B16F10 cells, MTT assays were conducted. B16F10 cells were exposed to each compound at concentrations ranging from 2.5 to 40 μM and incubated for 72 h. Cell viability was considered unaffected if it remained at 90% or higher compared to the untreated group. The results indicated that 4,4′-DMC exhibited no cytotoxicity at concentrations below 20 μM, 3,4′-DMC at concentrations below 10 μM, and 4′,6′-DMC at concentrations below 5 μM (Figure 2).

**Figure 3:**
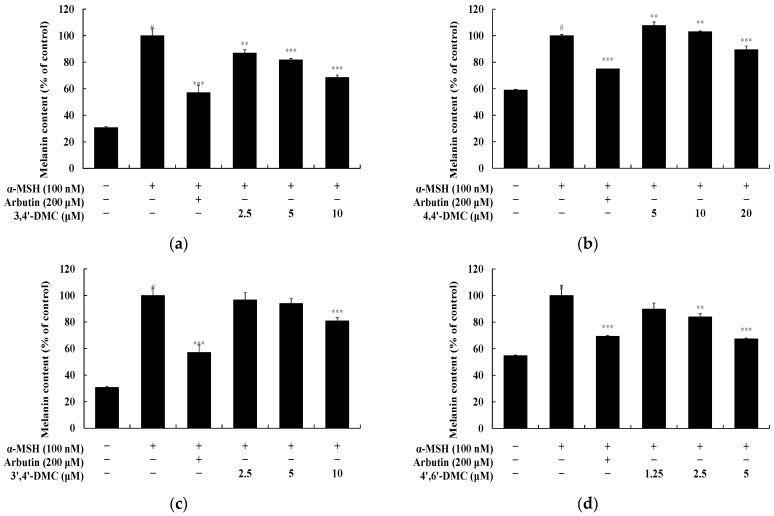
Effect of 2′-hydroxy-4′-methoxychalcone derivatives on melanin content of B16F10 cells. B16F10 cells were treated with 2′-hydroxy-4′-methoxychalcone derivatives for 72 h in the presence of α-MSH (100 nM) stimulation, with arbutin (200 μM) used as a positive control. The melanin content was measured for cells treated with (**a**) 3,4′-DMC, (**b**) 4,4′-DMC, (**c**) 3′,4′-DMC, and (**d**) 4′,6′-DMC. Data are expressed as the mean ± SD from three independent experiments. Statistical significance is indicated as # *p* < 0.001 compared to the untreated control, and ** *p* < 0.01, *** *p* < 0.001 compared to α-MSH alone.

**Figure 4:**
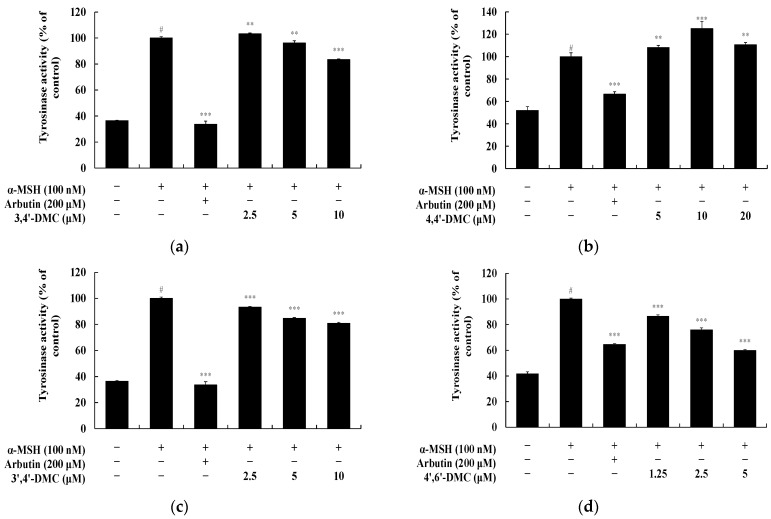
Effect of 2′-hydroxy-4′-methoxychalcone derivatives on tyrosinase activity of B16F10 cells. B16F10 cells were treated with 2′-hydroxy-4′-methoxychalcone derivatives for 72 h in the presence of α-MSH (100 nM) stimulation, with arbutin (200 μM) used as a positive control. Tyrosinase activity was measured for cells treated with (**a**) 3,4′-DMC, (**b**) 4,4′-DMC, (**c**) 3′,4′-DMC, and (**d**) 4′,6′-DMC. Data are expressed as the mean ± SD from three independent experiments. Statistical significance is indicated as # *p* < 0.001 compared to the untreated control, and ** *p* < 0.01, *** *p* < 0.001 compared to α-MSH alone.

To explore the impact of 2′-hydroxy-4′-methoxychalcone derivatives on melanin synthesis and tyrosinase activity in B16F10 cells at non-cytotoxic concentrations, melanin content and tyrosinase activity were assessed. B16F10 cells were exposed to varying concentrations of each compound and cultured for 72 h. α-MSH (100 nM) served as a negative control, while arbutin (200 μM) acted as a positive control. Specifically, at 5 μM, 4′,6′-DMC reduced melanogenesis by approximately 32.58%, 3,4′-DMC by approximately 18.25%, and 3′,4′-DMC by approximately 6.08%, whereas 4,4′-DMC increased melanogenesis by about 7.69% compared to α-MSH alone (Figure 3). Additionally, tyrosinase activity was inhibited by approximately 40.18% for 4′,6′-DMC, 3.69% for 3,4′-DMC, and 15.30% for 3′,4′-DMC, while it increased by approximately 8.16% for 4,4′-DMC compared to α-MSH alone (Figure 4). Consequently, 4′,6′-DMC, exhibiting the most potent anti-melanogenic activity, was further evaluated at concentrations below 5 μM, which demonstrated no cytotoxicity.

### 3.2. 4′,6′-DMC Regulated the Expression of Melanogenesis-Related Proteins in B16F10 Cells

**Figure 5:**
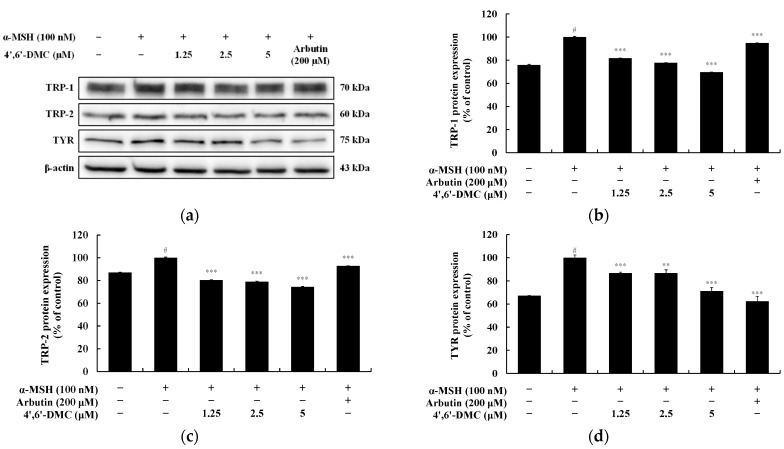
Effect of 4′,6′-DMC on protein expression of melanin synthesis-related enzymes in B16F10 cells. Cells were treated with 4′,6′-DMC (1.25, 2.5, and 5 μM) in combination with α-MSH (100 nM) for 24 h, with arbutin (200 μM) used as a positive control. The (**a**) protein abundance bands and expression levels of (**b**) TRP-1, (**c**) TRP-2, and (**d**) TYR were assessed. β-actin was used to ensure equal protein loading. Data are presented as the mean ± SD of three independent experiments, analyzed using ImageJ software (Version 1.54). Statistical significance is indicated as # *p* < 0.001 compared to the untreated control, and ** *p* < 0.01, *** *p* < 0.001 compared to α-MSH alone.

**Figure 6:**
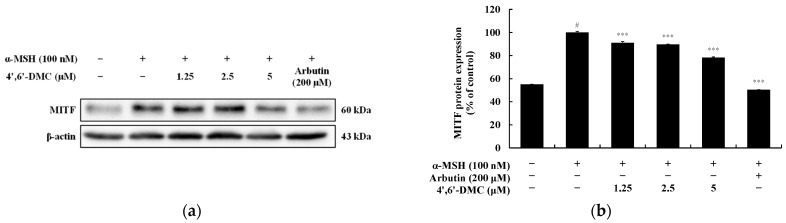
Effect of 4′,6′-DMC on protein expression of MITF in B16F10 cells. Cells were treated with 4′,6′-DMC (1.25, 2.5, and 5 μM) in combination with α-MSH (100 nM) for 24 h, with arbutin (200 μM) used as the positive control. The protein abundance bands (**a**) and expression levels (**b**) of MITF were assessed β-actin was used to ensure equal protein loading. Data are presented as the mean ± SD of three independent experiments, analyzed using ImageJ software. Statistical significance is indicated as # *p* < 0.001 compared to the untreated control, and *** *p* < 0.001 compared to α-MSH alone.

TYR, TRP-1, and TRP-2 are essential enzymes for melanin synthesis. MITF, a transcription factor for melanogenic enzymes, regulates the growth, differentiation, and function of melanocytes by activating enzymes such as TYR, TRP-1, and TRP-2. Hence, Western blots were conducted to explore the impact of 4′,6′-DMC on the expression of these melanogenesis-related proteins in α-MSH-stimulated B16F10 cells. The findings revealed a concentration-dependent reduction in the expression of TYR, TRP-1, and TRP-2 induced by α-MSH upon treatment with 4′,6′-DMC. Compared to the treatment with α-MSH alone (100 nM), the expression levels of TRP-1, TRP-2, and TYR were decreased by approximately 30.34%, 25.68%, and 28.82%, respectively, at 5 μM (Figure 5). To investigate the influence of MITF on these inhibitory effects of 4′,6′-DMC on melanogenic enzymes, the expression of MITF protein was examined. Consequently, the expression of MITF induced by α-MSH was significantly suppressed in a concentration-dependent manner, with 4′,6′-DMC reducing it by about 21.81% at 5 μM compared to the treatment with α-MSH alone (Figure 6).

### 3.3. 4′,6′-DMC Inhibits Melanogenesis through the GSK-3β/β-Catenin Pathway in B16F10 Cells

**Figure 7:**
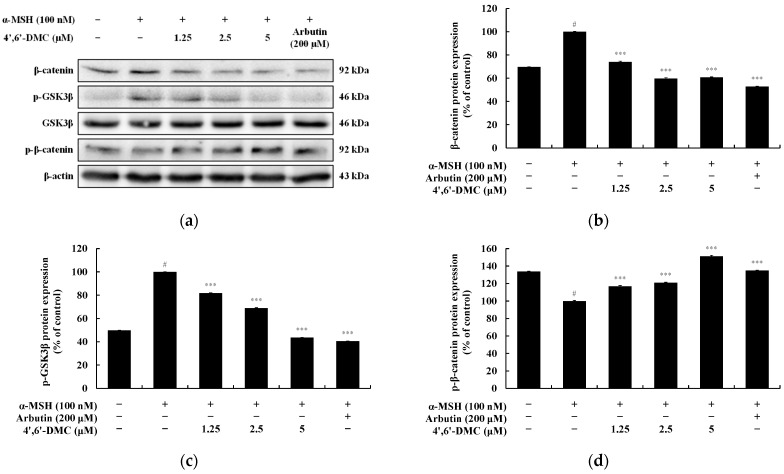
Effect of 4′,6′-DMC on protein expression of Wnt/β-catenin signaling pathways in B16F10 cells. Cells were treated with 4′,6′-DMC (1.25, 2.5, and 5 μM) in combination with α-MSH (100 nM) for 24 h and arbutin (200 μM) was used as the positive control. (**a**) Protein abundance bands and protein expression of (**b**) β-catenin, (**c**) p-GSK3β, and (**d**) p-β-catenin. β-actin was used to confirm equal amounts of protein loading. Data are presented as the mean ± SD of single triplicate experiments using ImageJ software. # *p* < 0.001 vs. untreated control. *** *p* < 0.001 vs. α-MSH alone.

In the Wnt/β-catenin signaling pathway, the membrane receptor protein Frizzled binds and interacts with Wnt ligands. GSK3β promotes the accumulation of β-catenin in the cytoplasm by phosphorylating and inactivating it at the serine 9 residue. Subsequently, the accumulated β-catenin translocates to the nucleus, ultimately enhancing the expression of MITF. Moreover, GSK3β phosphorylates β-catenin at tyrosine 216, inducing its ubiquitination and degradation, thereby reducing MITF expression. Upon investigating whether 4′,6′-DMC inhibits melanogenesis through the Wnt/β-catenin pathway, it was observed that 4′,6′-DMC significantly reduced α-MSH-induced β-catenin expression and GSK3β phosphorylation in a concentration-dependent manner. Additionally, β-catenin phosphorylation induced by α-MSH increased in a concentration-dependent manner. Compared to the treatment with α-MSH alone (100 nM), the expression of β-catenin and phosphorylated GSK3β decreased by approximately 39.37% and 56.36%, respectively, while phosphorylated β-catenin expression increased by approximately 51.12% at 5 μM (Figure 7).

### 3.4. 4′,6′-DMC Inhibits Melanogenesis through the PI3K/Akt Pathway in B16F10 Cells

**Figure 8:**
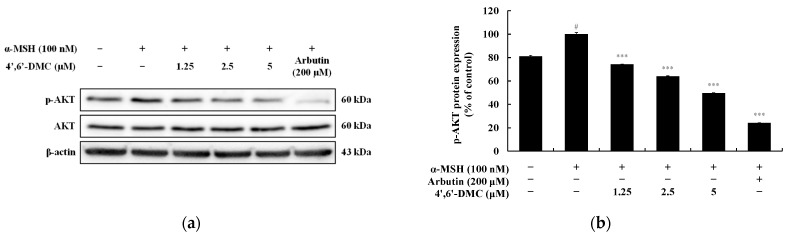
Effect of 4′,6′-DMC on protein expression of PI3K/AKT signaling pathways in B16F10 cells. Cells were treated with 4′,6′-DMC (1.25, 2.5, and 5 μM) in combination with α-MSH (100 nM) for 4 h and arbutin (200 μM) was used as the positive control. (**a**) Protein abundance bands and protein expression of (**b**) p-AKT. β-actin was used to confirm equal amounts of protein loading. Data are presented as the mean ± SD of single triplicate experiments using ImageJ software. # *p* < 0.001 vs. untreated control. *** *p* < 0.001 vs. α-MSH alone.

In the PI3K/AKT pathway, AKT is believed to phosphorylate GSK3β at serine 9, leading to its inactivation and subsequent inhibition of β-catenin degradation. When investigating whether 4′,6′-DMC inhibits melanogenesis through the PI3K/AKT pathway in B16F10 cells, it was observed that 4′,6′-DMC significantly reduced the phosphorylation of AKT induced by α-MSH. Compared to the treatment with α-MSH alone (100 nM), the expression of phosphorylated AKT at 5 μM was reduced by approximately 50.28% (Figure 8).

### 3.5. 4′,6′-DMC Inhibits Melanogenesis through the MAPK Pathway in B16F10 Cells

**Figure 9:**
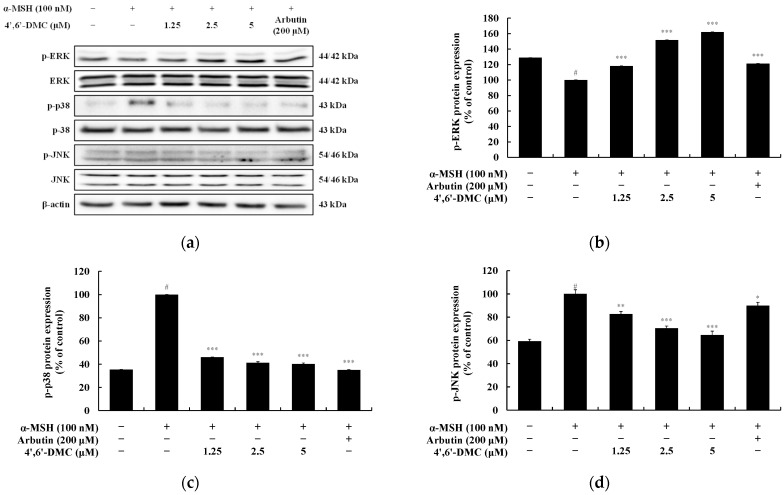
Effect of 4′,6′-DMC on protein expression of MAPK signaling pathways in B16F10 cells. Cells were treated with 4′,6′-DMC (1.25, 2.5, and 5 μM) in combination with α-MSH (100 nM) for 4 h and arbutin (200 μM) was used as the positive control. (**a**) Protein abundance bands and protein expression of (**b**) p-ERK, (**c**) p-p38, and (**d**) p-JNK. β-actin was used to confirm equal amounts of protein loading. Data are presented as the mean ± SD of single triplicate experiments using ImageJ software. # *p* < 0.001 vs. untreated control. * *p* < 0.1, ** *p* < 0.01, *** *p* < 0.001 vs. α-MSH alone.

In the MAPK pathway, the phosphorylation of ERK inhibits melanogenesis by reducing MITF expression. Conversely, phosphorylation of p38 and JNK enhances melanogenesis by increasing MITF expression. Upon investigating whether 4′,6′-DMC inhibits melanogenesis via the MAPK pathway, it was observed that phosphorylation of ERK (a negative signaling pathway) significantly increased with rising concentrations of 4′,6′-DMC. Conversely, phosphorylation of p38 and JNK (positive signaling pathways) significantly decreased. Compared to treatment with α-MSH alone (100 nM), 5 μM of 4′,6′-DMC increased phosphorylated ERK expression by approximately 62.18% and decreased phosphorylated p38 and JNK expression by approximately 59.71% and 35.34%, respectively (Figure 9).

### 3.6. 4′,6′-DMC Inhibits Melanogenesis through the cAMP/PKA Pathway in B16F10 Cells

**Figure 10:**
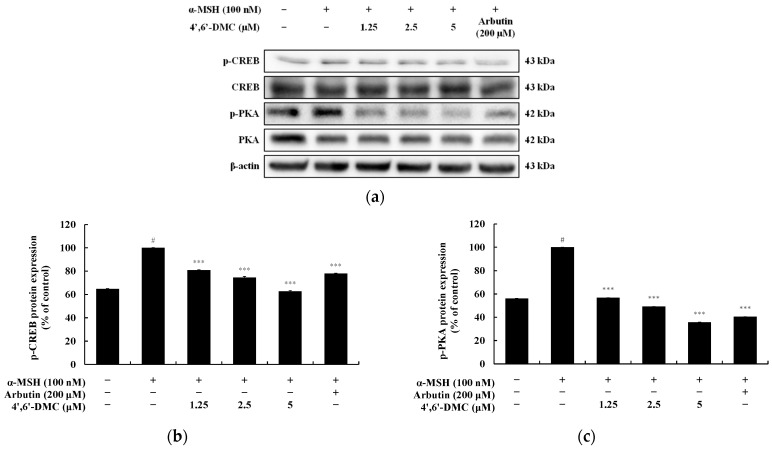
Effect of 4′,6′-DMC on protein expression of cAMP/PKA signaling pathways in B16F10 cells. Cells were treated with 4′,6′-DMC (1.25, 2.5, and 5 μM) in combination with α-MSH (100 nM) for 24 h and arbutin (200 μM) was used as the positive control. (**a**) Protein abundance bands and protein expression of (**b**) p-CREB and (**c**) p-PKA. β-actin was used to confirm equal amounts of protein loading. Data are presented as the mean ± SD of single triplicate experiments using ImageJ software. # *p* < 0.001 vs. untreated control. *** *p* < 0.001 vs. α-MSH alone.

In the cAMP/PKA pathway, activation of MC1R by α-MSH leads to intracellular cAMP accumulation. Subsequently, PKA undergoes phosphorylation and translocates to the nucleus due to the increased cAMP levels. This event triggers the phosphorylation and activation of CREB, thereby enhancing the transcription of MITF. To investigate whether 4′,6′-DMC inhibits melanogenesis through the PKA/CREB pathway, we examined the effect of 4′,6′-DMC on α-MSH-induced phosphorylation of CREB and PKA. The results showed a concentration-dependent reduction in phosphorylation levels upon treatment with 4′,6′-DMC. Compared to the treatment with α-MSH alone (100 nM), the expression of phosphorylated CREB and PKA decreased by approximately 37.27% and 64.25%, respectively, at 5 μM (Figure 10).

### 3.7. 2′-Hydroxy-4′-Methoxychalcone Derivatives Inhibited the Nitric Oxide Production in RAW 264.7 Cells

**Figure 11:**
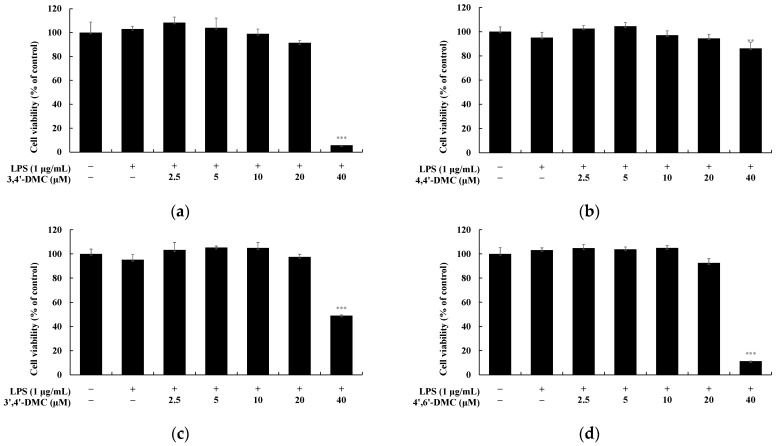
Cell viability of 2′-hydroxy-4′-methoxychalcone derivatives in RAW 264.7 cells. The cells were treated with 2.5, 5, 10, 20, and 40 μM 2′-hydroxy-4′-methoxychalcone derivatives for 24 h. Cell viability of RAW 264.7 cells subjected to (**a**) 3,4′-DMC, (**b**) 4,4,’-DMC, (**c**) 3′,4′-DMC, and (**d**) 4′,6′-DMC was measured via MTT assay. Results are expressed as the mean ± SD of three independent experiments. Statistical significance is indicated as ** *p* < 0.001, *** *p* < 0.001 compared with the untreated control.

MTT assays were conducted to determine the concentration range at which 2′-hydroxy-4′-methoxychalcone derivatives do not induce cytotoxicity in RAW 264.7 cells. Cells were exposed to each compound at concentrations ranging from 2.5 to 40 μM and incubated for 24 h. Cell viability was considered unaffected if it remained at 90% or higher compared to the untreated group. The results indicated that 3,4′-DMC, 4,4′-DMC, 3′,4′-DMC, and 4′,6′-DMC exhibited no cytotoxicity at concentrations below 20 μM (Figure 11).

**Figure 12:**
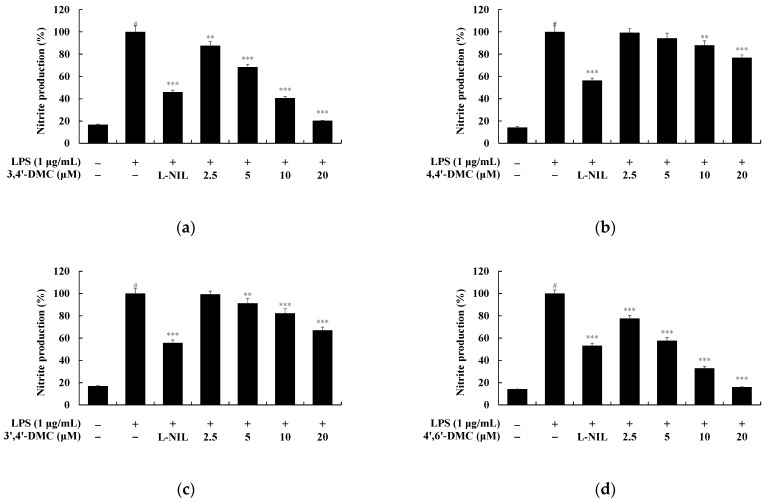
Effect of 2′-hydroxy-4′-methoxychalcone derivatives on nitric oxide (NO) production in RAW 264.7 cells. 2′-hydroxy-4′-methoxychalcone derivatives were applied to 264.7 cells for 24 h in the presence of LPS (1 μg/mL) stimulation, and L-NIL (40 μM) served as a positive control. NO production of RAW 264.7 cells treated with (**a**) 3,4′-DMC, (**b**) 4,4′-DMC, (**c**) 3′,4′-DMC and (**d**) 4′,6′-DMC. The results are expressed as the mean ± standard deviation from three independent experiments. The statistical significance is denoted as follows: # *p* < 0.001 vs. untreated control, ** *p* < 0.01, *** *p* < 0.001 vs. LPS alone.

To investigate the impact of 2′-hydroxy-4′-methoxychalcone derivatives on NO production in RAW 264.7 cells at non-cytotoxic concentrations, NO production was assessed. RAW 264.7 cells were exposed to various concentrations of each compound for 24 h, with LPS (1 μg/mL) serving as a negative control and L-NIL (40 μM) as a positive control. Specifically, at 20 μM, 4′,6′-DMC inhibited NO production by approximately 83.95%, 3′,4′-DMC by approximately 79.75%, 3′,4′-DMC by approximately 33.00%, and 4,4′-DMC by approximately 23.10% compared to LPS alone. Additionally, tyrosinase activity increased by about 40.18% for 4′,6′-DMC, 3.69% for 3′,4′-DMC, 15.30% for 3′,4′-DMC, and 8.16% for 4,4′-DMC compared to α-MSH alone (Figure 12). Therefore, 4′,6′-DMC, exhibiting the strongest anti-inflammatory activity, was further evaluated at concentrations below 20 μM, which demonstrated no cytotoxicity.

### 3.8. 4′,6′-DMC Inhibited the Production of Pro-Inflammatory Cytokines in RAW 264.7 Cells

**Figure 13:**
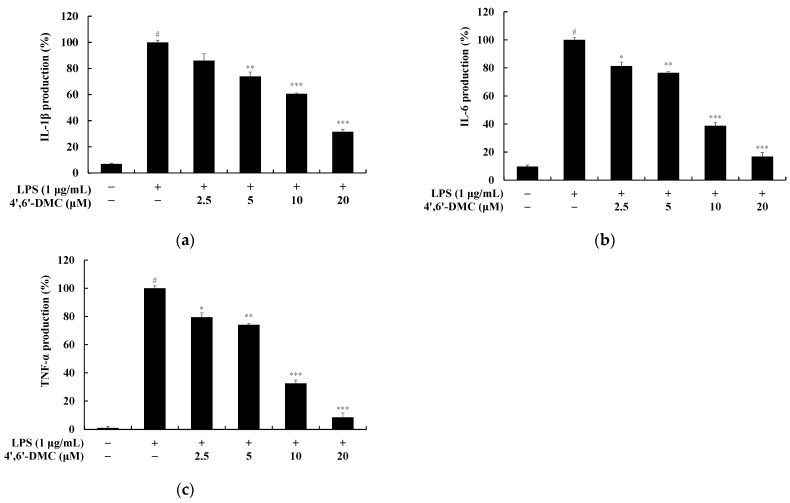
The impact of 4′,6′-DMC on the production of pro-inflammatory cytokines in RAW 264.7 macrophages. Cells were exposed to 4′,6′-DMC at concentrations of 2.5, 5, 10, and 20 μM for 24 h in the presence of LPS (1 μg/mL) stimulation. The production of pro-inflammatory cytokines, including IL-1β (**a**), IL-6 (**b**), and TNF-α (**c**), was assessed using an ELISA kit. The data are presented as the mean ± standard deviation from triplicate experiments. The statistical significance is denoted as follows: # *p* < 0.001 vs. untreated control, * *p* < 0.1, ** *p* < 0.01, *** *p* < 0.001 vs. LPS alone.

Cytokines, proteins secreted by immune cells, regulate inflammatory responses by modulating the activity, proliferation, and differentiation of immune cells. Upon LPS stimulation, macrophages activate multiple signaling pathways, resulting in the production of various inflammatory mediators and pro-inflammatory cytokines. To assess whether the sample inhibits the production of interleukin (IL)-1β, IL-6, and tumor necrosis factor (TNF)-α in LPS-induced RAW 264.7 cells, the production was measured using an ELISA kit. RAW 264.7 macrophages were treated with various concentrations (2.5–20 μM) of 4′,6′-DMC and cultured for 24 h. The extent of inhibition was then examined compared to the treatment with LPS alone (1 μg/mL). The results indicate that at 20 μM, 4′,6′-DMC inhibited the production of IL-6, IL-1β, and TNF-α by approximately 83.12%, 68.52%, and 91.64%, respectively, compared to the LPS alone treatment group (Figure 13).

### 3.9. 4′,6′-DMC Inhibited the Expression of iNOS and COX-2 Proteins in RAW 264.7 Cells

**Figure 14:**
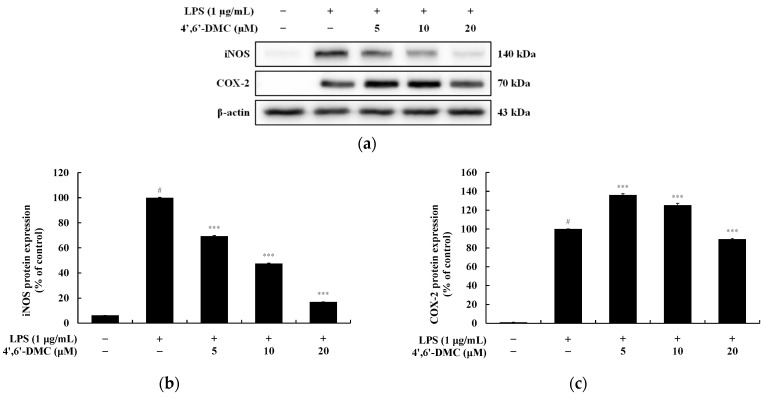
The impact of 4′,6′-DMC on the protein expression of iNOS and COX-2 in RAW 264.7 macrophages. RAW264.7 cells were treated with 4′,6′-DMC (5, 10, and 20 μM) in combination with LPS (1 μg/mL) for 24 h. (**a**) Protein abundance bands and protein expression of (**b**) iNOS and (**c**) COX-2. β-actin was utilized to verify consistent protein loading. The data are expressed as the mean ± standard deviation from individual triplicate experiments, analyzed using ImageJ software. Statistical significance is indicated as follows: # *p* < 0.001 vs. untreated control, *** *p* < 0.001 vs. LPS alone.

To explore whether the suppression of iNOS and COX-2 expression in RAW 264.7 cells led to a decrease in NO and PGE_2_ production, Western blots were conducted. The results indicate that 4′,6′-DMC suppressed the expression of iNOS and COX-2 induced by LPS in a concentration-dependent manner. Compared to the treatment with LPS alone (1 μg/mL), it reduced iNOS expression by 83.15% and COX-2 expression by 10.8% at 20 μM (Figure 14).

### 3.10. 4′,6′-DMC Inhibited Inflammation in RAW 264.7 Cells through the MAPK Signaling Pathway

**Figure 15:**
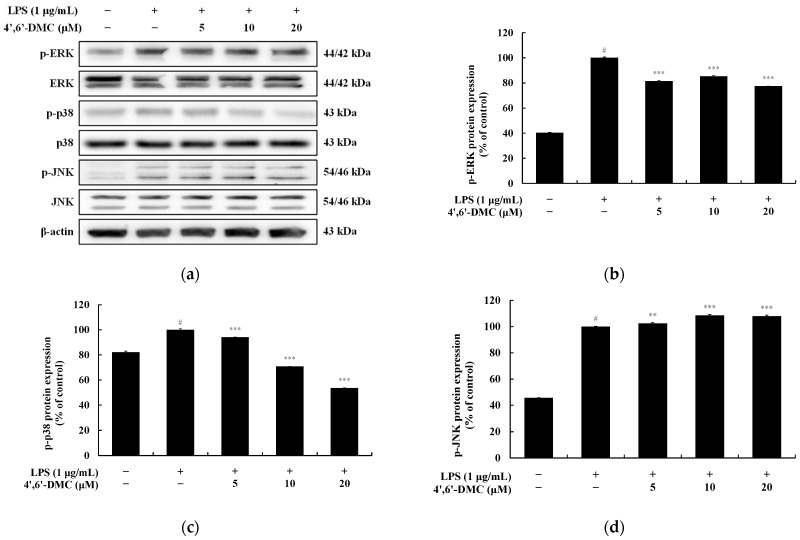
Effect of 4′,6′-DMC on the protein expression of MAPK signaling pathways. RAW 264.7 cells were treated with 4′,6′-DMC in combination with LPS for 20 min. (**a**) Protein abundance bands and protein expression of (**b**) p-ERK, (**c**) p-p38, and (**d**) p-JNK. β-actin was utilized to verify consistent protein loading. The data are expressed as the mean ± standard deviation from individual triplicate experiments, analyzed using ImageJ software. Statistical significance is indicated as follows: # *p* < 0.001 vs. untreated control, ** *p* < 0.01, *** *p* < 0.001 vs. LPS alone.

LPS stimulates TLR4 on the surface of macrophages, activating it through the induction of MAPK signaling pathway phosphorylation. This leads to an upsurge in the production of various inflammatory mediators and pro-inflammatory cytokines. To probe whether the inhibition of pro-inflammatory cytokine (IL-1β, IL-6, and TNF-α) and mediators (NO and PGE_2_) production by 4′,6′-DMC in LPS-stimulated RAW 264.7 cells is mediated via the MAPK signaling pathway, Western blots were conducted. The results indicate that 4′,6′-DMC inhibited LPS-stimulated phosphorylation of ERK and p38 in a concentration-dependent manner. Compared to the treatment with LPS alone (1 μg/mL), phosphorylated ERK and p38 expression was reduced by approximately 22.54% and 46.43% at 20 μM, respectively (Figure 15).

### 3.11. 4′,6′-DMC Repressed Inflammation in RAW 264.7 Cells through NF-kB Signaling Pathways

**Figure 16:**
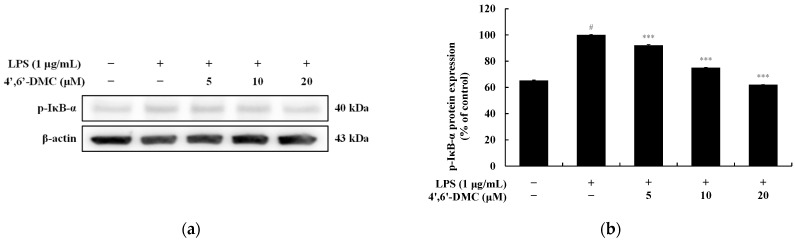
Effect of 4′,6′-DMC on the protein expression of NF-κB signaling pathways. RAW 264.7 cells were treated with 4′,6′-DMC (5, 10, and 20 μM) in combination with LPS (1 μg/mL) for 20 min. (**a**) Protein abundance bands and protein expression of (**b**) p-IκB-α. β-actin was utilized to verify consistent protein loading. The data are expressed as the mean ± standard deviation from individual triplicate experiments, analyzed using ImageJ software. Statistical significance is indicated as follows: # *p* < 0.001 vs. untreated control, *** *p* < 0.001 vs. LPS alone.

**Figure 17:**
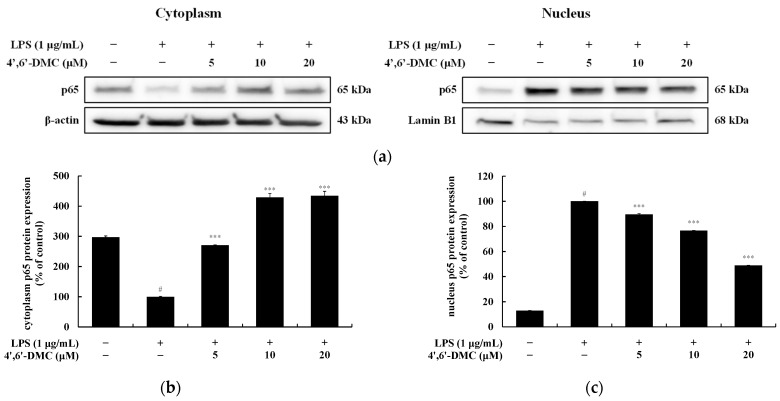
Effect of 4′,6′-DMC on the protein expression of NF-κB/p65 nuclear translocation. RAW 264.7 cells were treated with 4′,6′-DMC in combination with LPS for 15 min. (**a**) Protein abundance bands and protein expression of (**b**) cytoplasm p65 and (**c**) nucleus p65. β-actin was utilized to verify consistent protein loading. The data are expressed as the mean ± standard deviation from individual triplicate experiments, analyzed using ImageJ software. Statistical significance is indicated as follows: # *p* < 0.001 vs. untreated control, *** *p* < 0.001 vs. LPS alone.

NF-κB remains in an inactive state in the cytoplasm due to its association with IκB-α. Upon LPS stimulation in macrophages, IκB-α undergoes phosphorylation and degradation, leading to NF-κB activation. Activated NF-κB then translocates from the cytoplasm to the nucleus, where it triggers the expression of various proinflammatory cytokines and inflammatory mediators, including iNOS and COX-2. To investigate whether the inhibition of inflammatory mediators and pro-inflammatory cytokine production by 4′,6′-DMC in LPS-stimulated RAW 264.7 cells is mediated through the NF-κB pathway, Western blots were conducted. The results indicate that 4′,6′-DMC inhibited LPS-induced phosphorylation of IκB-α in a concentration-dependent manner, with phosphorylated IκB-α expression decreasing by approximately 37.98% at 20 μM compared to the treatment with LPS alone (1 μg/mL) (Figure 16). Subsequently, the translocation of NF-κB/p65 from the cytoplasm to the nucleus was assessed. It was observed that 4′,6′-DMC increased p65 production in the cytoplasm in a concentration-dependent manner, while significantly inhibiting p65 production in the nucleus. Specifically, compared to LPS alone (1 μg/mL), 20 μM 4′,6′-DMC increased p65 expression in the cytoplasm by approximately 334.41% and decreased p65 expression in the nucleus by approximately 51.11% (Figure 17).

### 3.12. 4′,6′-DMC Is Safe for Human Skin

The purpose of the study was to assess the primary irritant effect of two concentrations (5 μM and 10 μM) of 4′,6′-DMC dissolved in squalene on human skin, following PCPC guidelines and Dermapro Inc.’s standard operating procedure. The patch test involved 34 female subjects meeting inclusion and exclusion criteria, with a mean age of 41.56 ± 7.37 years. The test material (20 μL) was applied to cleaned test sites on the subjects’ backs for 24 h, and evaluations were conducted 20 min and 24 h after removal. Primary skin irritation response was assessed according to PCPC Guidelines (Table 1). The results indicated that 4′,6′-DMC exhibited hypoallergenic properties concerning primary skin irritation (Table 2).

## 4. Discussion

Chalcones are abundant in edible and medicinal plants, and their derivatives have been studied for various biological activities [46]. Despite the development of numerous pharmacological properties in chalcone derivatives, the anti-melanogenic and anti-inflammatory effects of 2′-hydroxy-4′-methoxychalcone have not been fully investigated. Therefore, we aimed to explore the biological activities of structurally different 2′-hydroxy-4′-methoxychalcone derivatives in B16F10 cells and RAW 264.7 cells.

Melanin, a pigment found in the skin, hair, and eyes, regulates the skin’s immune response by absorbing harmful UV rays and protecting the body from UV radiation. However, when melanin is overproduced and accumulates in the skin, it can cause hyperpigmentation. The TYR enzyme catalyzes melanin synthesis, with TRP-1 and TRP-2 proteins involved in producing eumelanin, which gives a blackish-brown color. The transcription factor MITF activates the expression of these melanogenic genes, inducing protein expression and regulating melanogenesis. Therefore, blocking MITF-related signaling pathways is considered an important strategy for developing melanogenesis inhibitors [54,55,56]. 

TYR inhibitors are frequently employed for treating hyperpigmentation. Mushroom TYR stands as the sole commercially available form, boasting the closest resemblance to mammalian TYR. Although kojic acid, a 3-hydroxy-4-pyrone compound, remains the most extensively researched TYR inhibitor, its usage has been banned in numerous countries due to adverse effects including dermatitis, carcinogenicity, hepatotoxicity, and unspecified mechanisms. Furthermore, conventional tyrosinase inhibitors utilized in cosmetics, such as hydroquinone and its glycoside (arbutin), have also been linked to side effects such as erythema, vitiligo, skin allergies, dermatitis, acne, flaky skin, and dryness. Hence, the development of natural whitening agents characterized by superior permeability, minimal irritation, and heightened safety has emerged as a paramount concern within the realm of tyrosinase inhibitors [57,58,59,60,61,62]. 

For this reason, we checked whether the 2′-hydroxy-4′-methoxycalcone derivatives inhibited melanogenesis in this study. Additionally, we identified the biological differences between the 2′-hydroxy-4′-methoxycalcone derivatives based on structural variances and compared their biological characteristics depending on the location of the methoxy group. In terms of structure and melanin synthesis, we found that 4′,6′-DMC, with a methoxy group at the 6′-position of 2′-hydroxy-4′-methoxychalcone, exhibited the best melanogenesis inhibitory activity in B16F10 cells stimulated with α-MSH. Next, we investigated the different signaling pathways involved in melanogenesis affected by 4′,6′-DMC. As shown in Figure 5, the expression of melanogenic enzymes such as TYR, TRP-1, TRP-2, and their transcription factor MITF was decreased by 4′,6′-DMC. This suggests that 4′,6′-DMC inhibits melanogenesis by down-regulating the expression levels of the transcription factor MITF and the melanogenic enzymes. 

Furthermore, as shown in Figure 7, we found that 4′,6′-DMC significantly decreased the expression of β-catenin and the phosphorylation of GSK3β induced by α-MSH in a concentration-dependent manner. Meanwhile, the phosphorylation of β-catenin increased in a concentration-dependent manner, which down-regulated the expression level of the transcriptional regulator MITF. This suggests that 4′,6′-DMC inhibits the expression of melanogenic enzymes through the Wnt/β-catenin signaling pathway.

Additionally, we confirmed whether 4′,6′-DMC is involved in the PI3K/AKT and MAPK pathways, which are crucial signaling pathways in melanogenesis. As shown in Figure 8 and Figure 9, the phosphorylation of AKT, closely related to GSK3β, was significantly decreased, confirming that melanogenesis is inhibited through the PI3K/AKT pathway. The phosphorylation of ERK, a negative regulator in melanogenesis, increased, while the phosphorylation of p38 and JNK, which are positive regulators, decreased. Therefore, it can be concluded that 4′,6′-DMC inhibits melanogenesis through the MAPK pathways by downregulating the expression level of MITF, a transcriptional regulator. 

Finally, the phosphorylation of CREB and PKA induced by α-MSH was reduced by 4′,6′-DMC in a concentration-dependent manner, suggesting that 4′,6′-DMC also inhibits melanogenesis via the cAMP/PKA pathway. Based on these results, 4′,6′-DMC decreased intracellular TYR activity and melanin content in B16F10 cells by downregulating MITF, PKA, CREB, p38, JNK, β-catenin, GSK3β, and AKT proteins, and upregulating ERK and p-β-catenin. Taken together, 4′,6′-DMC may be a promising candidate for the development of whitening agents and hyperpigmentation therapeutics. 

Inflammation is a beneficial biochemical reaction of the body to fight external harmful substances, but excessive inflammation can lead to inflammatory diseases in various organs, such as allergies, rheumatoid arthritis, atherosclerosis, and asthma. Nonsteroidal anti-inflammatory drugs (NSAIDs), which are commonly used, can cause various side effects, and serious clinical sequelae have been reported in some patients who have overdosed on NSAIDs. Consequently, many studies have been conducted to find safe and effective anti-inflammatory drugs. In this context, we investigated the anti-inflammatory efficacy of 2′-hydroxy-4′-methoxychalcone derivatives. As shown in Figure 12, among the four derivatives, 4′,6′-DMC, which has a methoxy group at position 6′, exhibited the best inhibitory effect on NO production. Therefore, we further investigated the production of pro-inflammatory cytokines and various signal transduction pathways involved in inflammation using 4′,6′-DMC. 

Next, we investigated the production of proinflammatory cytokines using an ELISA kit and confirmed that 4′,6′-DMC concentration-dependently suppressed the excessive induction of IL-1β, IL-6, and TNF-α by LPS, as depicted in Figure 14. Additionally, the expression of iNOS and COX-2 was reduced, indicating that 4′,6′-DMC could mitigate the production of proinflammatory mediators such as NO. Furthermore, through a Western blot experiment, we observed that 4′,6′-DMC inhibited the phosphorylation of ERK and p38, thereby down-regulating the production of NO and proinflammatory cytokines by suppressing the activation of the MAPK pathway (Figure 15). 

Additionally, we investigated the involvement of the NF-κB signaling pathway in the anti-inflammatory effect of 4′,6′-DMC. As illustrated in Figure 16 and Figure 17, 4′,6′-DMC concentration-dependently inhibited the phosphorylation of IκB-α induced by LPS. Moreover, 4′,6′-DMC increased the cytoplasmic production of p65 in a concentration-dependent manner, while significantly inhibiting its nuclear production. This finding suggests that 4′,6′-DMC attenuates inflammation by modulating the NF-κB signaling pathway and impeding nuclear translocation.

According to OECD guidelines (OECD 439), skin irritation is defined as reversible damage to the skin after the application of a test substance. Understanding the potential for skin irritation (hazardousness) of topically applied formulations is considered critical for safety assessment. Therefore, human skin irritation tests were performed to evaluate the application of 4′,6′-DMC as a cosmeceutical or ointment. As a result, the human skin irritation study evaluated the potential for the topical application of 4′,6′-DMC and found no use-related side effects (Table 2). Overall, these findings suggest that 4′,6′-DMC holds promise for the treatment of inflammatory skin conditions such as atopic dermatitis or acne, as well as for the development of cosmeceuticals or ointments.

## Figures and Tables

**Table 1:** Grading system for skin primary irritation test.

Grade	Description of Clinical Observation
+1	Slight erythema
+2	Moderate erythema, possibly with barely perceptible edema at the margin, papules may be present
+3	Moderate erythema, with generalized edema
+4	Severe erythema with severe edema, with or without vesicles
+5	Severe reaction spread beyond the area of the patch

**Table 2:** Results of human skin primary irritation test (*n* = 34).

No	Test Samples	No. of Responder	20 min after Patch Removal				24 h after Patch Removal				Reaction Grade (R)
			+1	+2	+3	+4	+1	+2	+3	+4	
1	4′,6′-DMC(10 μM)	0	0	0	0	0	0	0	0	0	0
2	4′,6′-DMC(5 μM)	0	0	0	0	0	1	0	0	0	0

## Data Availability

The original contributions presented in the study are included in the article, further inquiries can be directed to the corresponding author.
